# Trace metal element pollution of soil and water resources caused by small-scale metallic ore mining activities: a case study from a sphalerite mine in North China

**DOI:** 10.1007/s11356-019-05703-z

**Published:** 2019-06-25

**Authors:** Jingzhao Lu, Hongwei Lu, Kaiwen Lei, Weipeng Wang, Yanlong Guan

**Affiliations:** 1 School of Renewable Energy, North China Electric Power University, Beijing, 102206 China; 2 Key Laboratory of Water Cycle and Related Land Surface Processes, Institute of Geographic Science and Natural Resources Research, Chinese Academy of Science, Beijing, 100101 China

**Keywords:** Trace metal elements, Soil and water, Ecological risk, Human health risk

## Abstract

Trace metal element contamination in mining areas is always a huge environmental challenge for the global mining industry. In this study, an abandoned sphalerite mine near the Yanshan Mountains was selected as subject to evaluate the soil and water contamination caused by small-scale mining. The results show that (1) Pearson correlation matrix and principal component analysis (PCA) results reveal that Zn, Cu, Cd, and Pb were greatly affected by the operation of mines, especially mineral tailings. The contents of trace metal elements decrease with the increase of the distance from the mining area. Zinc, Pb, and Cd were discovered in almost all soil samples, and Zn accounted for about 80% of pollution of the topsoil. (2) The trace element pollution levels in the topsoil of the three villages were ranked as follows: Cd > Cu > Pb~Zn. The potential ecological risk of farmland around the mine ranges from lower to higher, with Cd being the most harmful. (3) Human health risk assessment results show that trace elements in the mining area pose obvious non-carcinogenic health risks to children while the risks to adults are not equally obvious. The carcinogenic risk of Cd and Cr is within a safe range and does not pose an obvious cancer risk to the population.

**Electronic supplementary material:**

The online version of this article (10.1007/s11356-019-05703-z) contains supplementary material, which is available to authorized users.

## Introduction

Trace element pollution in mining areas is always a huge environmental challenge for the global mining industry (Bhuiyan et al. 2010). With the weathering of pyrite waste, a large amount of harmful trace elements enter the soil, water, and surrounding environment (Chopin and Alloway 2007; Obiora et al. 2016). Pollution hazards of some trace metal elements could be acute due to the serious toxicity that they present even in low concentrations, such as zinc (Zn), cadmium (Cd), lead (Pb), and mercury (Hg) (Belén et al. 2012; Carvalho et al. 2011). Their sustainable and long-term bioaccumulation poses a huge threat to human health (Pandey et al. 2016; Rodríguez et al. 2009). Understanding the sources and relevant characteristics of trace metals can provide useful information for decision makers on the purpose of effectively developing mine remediation policies (Chen et al. 2017; Zieliński 2016). Previously, many studies emphasized the trace element contamination and ecological risks caused by large-scale mining operations (Ogunkunle and Fatoba 2014). Especially, most of them analyzed the differences of soils, waters surrounding the mining area with aspects like water composition, geological conditions, mineralization type, rock style, and microbial distributions in soils. For example, Kamunda et al. (2016) found that the biggest Cu-Mo mining area in Armenia produced high levels of soil contamination, poising human non-carcinogenic risk. Chabukdhara et al. (2017) reported that toxic metals such as Cu, Zn, Cd, and Hg are associated with anthropogenic contributors caused by mining activities in the Lower Pra Basin of Ghana (in Africa), while As, Se, and Pb could be related with both anthropogenic and natural geochemical processes. Acid mine drainage (AMD) is an acidic mineral solution that flows out of a mine. The phenomenon is spontaneous and involves metal sulfides (type “M” Sx) with no economic value in mining. The oxidation of pyrite (FeS_2_) plays a central role in the production of acid mine drainage. This mineral represents most of the sulfide phases (Moyé et al. 2017). This oxidation is a series of precipitation, absorption, complexation, chelation, and redox progress (Belén et al. 2012; Blowes et al. 2003; Marquez et al. 2018). Metals/metalloids in AMD are either absorbed by plants or deposited in soil and sludge, which consequently affect the growth of animals and plants (Intamat et al. 2016; Islam et al. 2015).

Although the contamination and ecological risks caused by large-scale mining activities had been well recorded worldwide, little importance had been attached to those caused by small-scale mines (Carkovic et al. 2016; Ding et al. 2018; Gevorg et al. 2018). Especially, illegal mining activities have resulted in different trace metal element pollution in soils, water, and the atmosphere. Among others, a considerable number of small mines are situated in the Yanshan Mountain of north China, which mainly consist of chalcopyrite (CuFeS_2_), sphalerite (ZnS), galena (PbS), and other mineral resources (Cope et al. 2010; Hu et al. 2010). Due to inconvenient transportation, complex mountainous terrain, and sparse population, it is difficult for these mining areas to attract large mining companies. Therefore, local developers can only have conducted several mining operations in a primitive manner and sell the ores directly to large mining companies for the following flotation treatment. Compared with state-owned mines with complete qualifications and regulations, those small mines are located in remote areas, lacking of governmental regulation and environmental awareness (Gevorg et al. 2018). The drainage generated during mining operation is discharged in a random way. Unfortunately, such small-scale mining activities are supported by local governments and the public, even if they pose a serious threat to the local environment. In addition, the owners of the small mines would stop mining without taking any protective measures once the economy goes down or the capital is insufficient. Therefore, those abandoned sites remain the sources of contamination even after many years of closure. To our knowledge, no work has been reported on the source of trace metal element pollution in the Yanshan Mountains, especially the role that arbitrary waste disposal and small-scale mining activities plays on affecting the soil and water quality, in a direct or indirect manner.

Herein, we present the study of the water and soil contamination caused by small-scale mining in an abandoned sphalerite mine area in the Yanshan Mountains. First, multivariate statistical analysis, Pearson correlation matrix, and principal component analysis (PCA) were proposed to recognize the features of trace elements in soil and water. Then, the pollution index method of geostatistics was employed to assess the pollution levels of trace elements in soil and analyze corresponding contents in the tailing, soil and water samples of the mining area. Finally, the potential ecological and human health risks of heavy metals caused by abandoned mines were also respectively assessed.

## Materials and methods

### Survey area and sample collection

**Fig. 1:**
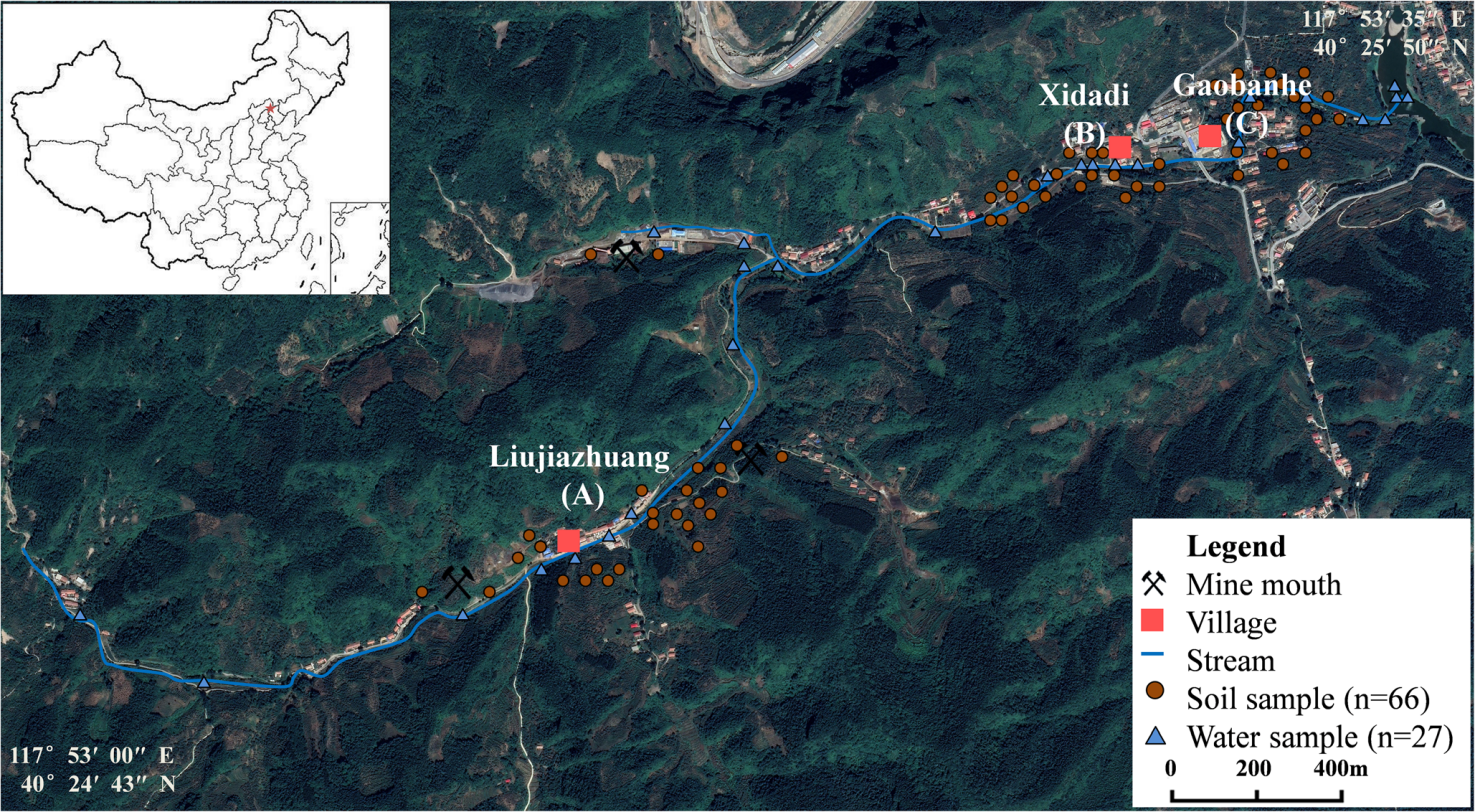
General setting of the sphalerite mining area and the sampling sites in the Yanshan Mountains

The sampling sites are located in an abandoned sphalerite mine area in Chengde, Hebei province (Fig. 1). The mining areas are distributed on different hillsides. Large-scale mining activities began in 2006 and most of the sites had been abandoned by 2012. Until now, only a few primitively operated mines are still open. There are lots of tailings, mine slags, along with some abandoned gravel for road building scattered on the hillsides.

The surveyed area is about 10 km^2^, including 5 km^2^ of mining area and a nearby farmland. All the mining sites are distributed on the gently sloping hillsides around the villages. There are three villages nearby: Liujiazhuang (A), Xidadi (B), and Gaobanhe (C). Villages B and C are located below the mining sites, whereas village A is close to the sites.

The climate is monsoon continental, with an annual average temperature of 7.5 °C and an average precipitation of 727.9 mm. Due to the mining operation, runoff caused by precipitation in the area causes erosion of the surface soil. A large amount of slags enter the surface runoff along with a large number of sediments into the downstream area. Meanwhile, the emission of mine drainage increased the concentration of trace elements in the topsoil and streams of the surrounding area. The main crop is corn and this is grown even on the very steep slopes of the study area. The drainage coming from different mining sites is directly discharged into two streams, which both pass by village A and merge into village B; the streams also reach the margin of field of village C. Furthermore, villages B and C are relatively further from the mining area and different in altitude (Fig. 1).

In September 2018, samples were collected from tailings, farmlands, bare land, and surface waters (including mine drainage) on the basis of the distribution of mines and plants and soil types. Eleven tailing samples were collected from the vicinity of the mining area, 6 samples were collected from the soils adjacent to the tailings, and 60 topsoil (0–40 cm) samples were gathered from 700 × 700 m grids in fields (Fig. 1). For the purpose of determining the background of trace elements in local farmland, a number of 9 topsoil samples were gathered from the farmland with a distance of 5–10 km located in the southeast of the mining area, an area unaffected by mining activities. Then, 3 drainage samples were collected from the source of drainage from the mine, and 2 water samples were collected at the source of the streams near the mine. Meanwhile, water samples (*n* = 22) were taken from streams that begin in the mining area and pass through the downstream farmland (Fig. 1). The tailings and the soil samples (300 g for each) were properly packed in poly bags. The water samples were put into 50-mL poly centrifuge tubes after being filtered through a cellulose acetate membrane with a thickness of 20 μm. After those steps, all of the aforementioned samples were delivered back to the Chinese Academy of Sciences for further analysis within 48 h.

### Chemical analysis

Chemical analysis of soil samples was conducted in the Physical and Chemical Analysis Center of the Institute of Geographic Sciences and Natural Resources Research, Chinese Academy of Sciences. After being air-dried, plant residues and stones were removed from soil and tailing samples. About 10 g of the dried samples was sieved through a 2-mm nylon sieve for later use and a portion of this (0.2 g) was digested in a mixed acid HNO_3_-HClO_4_-HF (1:1:1) solution (10 mL). After that, the samples of soils and tailings were heated at 120 °C on an electric hot plate to near dryness, and then re-dissolved with 10 mL (1% HNO_3_). Finally, ICP-MS (EDX4500P) was used to determine the contents of Fe, Mn, Zn, Pb, Cr, Cu, Hg, and Cd in all solid samples (Khalil et al. 2013).

The water samples (10 mL) were filtered through a 0.45-μm membrane filter and then acidified to pH < 2 using analytical grade nitric acid (1% HNO_3_). Later, Fe, Mn, Zn, Pb, Cr, Cu, Hg, and Cd were measured using ICP-MS (EDX4500P).

A quality assurance/quality check (QA/QC) program was established and implemented to ensure the production of validate results for all samples (i.e., soil, tailings, and water). The QA/QC process entailed instrument calibration and parallel sample experiments. The instrument was calibrated before analyzing the samples with a blank and appropriate calibration standard. Calibration standards of 1, 10, 50, and 100 μg/L were applied to calibrate the instrument and only *R*^2^ above 0.999 was accepted. The calibration was verified with a 10 μg/L and 50 μg/L standard followed by the analysis of the samples with appropriate internal standard (i.e., Fe, Mn, Zn, Pb, Cr, Cu, Hg, and Cd). The parallel sample experiments were performed in triplicate and data were reported as an averaged value with standard deviations (SD). Data was considered acceptable when percentage difference within triplicate samples and percent error were below 10%. The analytical values below detection limit (BDL) were managed on the basis of the EPA guideline. The accuracy of the analysis was tested by the analysis of the National Institute of Standards and Technology (NIST) water standards. In addition, several samples of soil, tailings, and water were taken for inter-laboratory analysis to further ensure the validity of the results.

### Statistical analysis

Pearson correlation matrixes, coupled with principal component analysis (PCA), were proposed to analyze the distribution of trace elements in surface soil (Jing et al. 2011). The correlation among the metals was used for identifying the source of trace elements. The statistical analysis was performed with SPSS 13.0.

The pollution index (PI) was calculated to evaluate the pollution grade of trace metal element in the topsoil, according to the following expression:

1 $$ \mathrm{PI}={C}_i/{S}_i $$

*C*_*i*_ is the measured concentration of trace elements in the soil (mg kg^−1^) and *S*_*i*_ is the reference concentration (mg kg^−1^). The *S*_*i*_ values of trace elements were determined on the basis of the class II standard (farmland soil, pH > 6.5) detailed in the National Standard for Soil Environment Quality Standard (SEPA 1995), shown in Table 1. According to the PI value, the degree of trace metal element pollution of surface soil is classified into mild (PI ≤ 1), moderate (1 < PI ≤ 3), or severe (PI > 3).

**Table 1:** Defining equations of daily intake via various exposure pathways

Classes	Functions	Calculation formula
Exposure pathway	Ingestion	${\mathrm{ADD}}_{\mathrm{ing}}=C\times \frac{\mathrm{CS}\times {R}_{\mathrm{ing}}\times \mathrm{EF}\times \mathrm{ED}}{\mathrm{BW}\times \mathrm{AT}}\times {10}^{-6}$
	Dermal contact	${\mathrm{ADD}}_{\mathrm{derm}}=\frac{C\times \mathrm{SL}\times \mathrm{SA}\times \mathrm{ABS}\times \mathrm{EF}\times \mathrm{ED}}{\mathrm{BW}\times \mathrm{AT}}\times {10}^{-6}$
	Diet	${\mathrm{ADD}}_{\mathrm{inh}}=\frac{C\times {R}_{\mathrm{inh}}\times \mathrm{EF}\times \mathrm{ED}}{\mathrm{BW}\times \mathrm{AT}\times \mathrm{PEF}}$

The Nemerow integrated pollution index (PI_N_) was used to assess the degree of pollution caused by different trace metal elements (Masaka et al. 2017), as follows:

2 $$ {\mathrm{PI}}_{\mathrm{N}}=\sqrt{\frac{{\mathrm{PI}}_{\mathrm{ave}}^2+{\mathrm{PI}}_{i\ \max}^{\kern3em 2}}{2}} $$

In the formula, PI_ave_ is the average of PI values of the considerable trace elements and PI_*i* max_ is the maximum of them. Surface pollution can be classified into 5 categories according to the PI_N_ values, as shown in Table S1.

Ecological risk index (RI), introduced by Hakanson (1980), was employed to evaluate the potential risks in the topsoil of abandoned mines caused by trace metal element (Chai et al. 2014; Lu et al. 2017). It is calculated as the sum of the *E*_*i*_ values for each trace metal element:

3 $$ \mathrm{RI}=\sum {E}_i $$

*E*_*i*_ is the single risk indexes for the trace metal element *i*, which is calculated from the measured concentration (*C*_*i*_) and the corresponding background concentration (*B*_*i*_):

4 $$ {E}_i={T}_i\frac{C_i}{B_i} $$

*T*_*i*_ is the toxicity response coefficient of the trace metal element *i*, and the *T*_*i*_ values of Cr, Cu, Zn, Hg, Cd, and Pb are respectively 2, 5, 1, 40, 30, and 5. *B*_*i*_ value can be also picked from the background concentration of trace metal elements in the topsoil of the Hebei province (SEPA 1990).

According to *E*_*i*_ and RI, the potential ecological risks of trace elements in soil fall into 5 categories, as shown in Table S2 (Ma et al. 2015).

Enrichment factor (EF), which can be used to identify the anthropogenic contribution relative to that of lithogenic sources for trace metal elements, is calculated as:

5 $$ \mathrm{EF}=\frac{C_n/{C}_{\mathrm{ref}}}{B_n/{B}_{\mathrm{ref}}} $$

where *C*_*n*_ and *C*_ref_ are the concentrations of target metal and reference element in the soil, respectively, while *B*_*n*_ and *B*_ref_ are the background concentrations of the target metal and reference element, respectively. The EF values of the target metals were calculated using Ti as the reference element, because the contents of Ti in the surface soils were not affected by anthropogenic sources. The concentrations of trace metal elements in the surface soils of Hebei province were used as the background values. The EF values can be classified into five levels to indicate the varying impact of human activity (Table S3). The trace metal elements are considered to be of natural origin when EF ≤ 1, whereas EF ≥ 1.5 are indicative of significant contribution from anthropogenic sources.

### Health risk assessment

The models for health risk assessment, divided into carcinogenic and non-carcinogenic types, are proposed by US EPA (Souza et al. 2017). Normally, there are three exposure pathways that human may be impacted by trace metal elements in soil: (1) direct ingestion of soil particles, (2) diet through the food chain, and (3) inhalation of soil particles (Akoto et al. 2018). The calculations for the daily exposure dose of contaminants via various exposure pathways are listed in Table 1 (US EPA 1991, 2002, 2011).

The non-carcinogenic risks from individual trace metal element can be calculated as follows:

6 $$ \mathrm{HI}=\sum {\mathrm{HQ}}_{\mathrm{i}}=\sum \frac{{\mathrm{ADD}}_{\mathrm{i}\mathrm{j}}}{{\mathrm{RfD}}_{\mathrm{i}\mathrm{j}}} $$

where the chronic hazard index (HI) is the sum of more than one hazard quotient for multiple substances or multiple exposure pathways, the non-carcinogenic hazard quotient (HQ) is the ratio of exposure to hazardous substances, ADD_ij_ is the chronic daily intake (mg kg^−1^ day^−1^), and RfD_ij_ is the chronic reference dose for the trace metal element *i* (mg kg^−1^ day^−1^). HI values > 1 mean that non-carcinogenic risk may occur, and when HI < 1, the reverse applies.

The carcinogenic risks from individual trace metal elements can be evaluated as follows:

7 $$ {R}_{\mathrm{Total}}=\sum {R}_i=\sum {\mathrm{LADD}}_{\mathrm{ij}}\times {\mathrm{SF}}_{\mathrm{ij}} $$

where LADD_ij_ is the chronic daily intake (mg kg^−1^ day^−1^) of substance *i* and SF_ij_ is the slope factor for substance *i* (mg kg^−1^ day^−1^). The acceptable or tolerable risk for regulatory purposes is within the range of 10^−6^ to 10^−4^ (US EPA 2011). In this paper, Pb, Cd, Cr, Zn, Cu, and Hg can pose chronic non-carcinogenic risks to humans, while only Cd and Cr can cause carcinogenic risk (US EPA 2002). Besides, the US EPA model merely renders the carcinogenic slope factor data for the inhalation pathway for Cd and Cr; therefore, this study only takes the exposure through the above pathway into consideration and leave out the cancer risks caused by trace metal elements through other pathways. The input parameters of health risk assessment models are listed in Table 2. Reference dose for non-carcinogenic metals and slope factors for carcinogenic metals are listed in Table 3.

**Table 2:** Exposure parameters for the health risk assessment models

Parameters	Meaning	Units	Values		Sources
			Child	Adults	
EF	Exposure frequency	day a^−1^	350		US EPA 2011
ED	Exposure duration	a	6	24	US EPA 2011
AT	Averaging time for non-carcinogens	day	ED × 365		US EPA 2002
AT_ca_	Averaging time for carcinogens	day	70 × 365		US EPA 2002
BW	Body weight	kg	15.9	56.8	US EPA 1991
*R*_ing_	Ingestion rate	mg day^−1^	200	100	US EPA 2011
*R*_inh_	Ingestion rate	m^3^ day^−1^	7.5	14.5	US EPA 2011
PEF	Particle emission factor	m^3^ kg^−1^	1.36 × 10^9^		USEPA 2002
SL	Adherence factor	mg cm^−2^ day^−1^	0.2	0.07	US EPA 2011
SA	Exposure skin area	cm^2^	1150	2145	US EPA 2011
ABS	Dermal absorption fraction	–	0.001		US EPA 2011

**Table 3:** Reference dose for non-carcinogenic metals and slope factors for carcinogenic metals

Program	Cd	Cr	Cu	Hg	Pb	Zn
RfD_ing_/[mg kg^−1^ day^−1^)]	1.00 × 10^−3^	3.00 × 10^−3^	4.00 × 10^−2^	3.00 × 10^−4^	3.50 × 10^−3^	0.3
RfD_inh_/[mg kg^−1^ day^−1^]	1.00 × 10^−3^	2.86 × 10^−5^	4.02 × 10^−2^	3.00 × 10^−4^	3.52 × 10^−3^	0.3
RfD_derm_/[mg kg^−1^ day^−1^]	1.00 × 10^−5^	6.00 × 10^−5^	1.20 × 10^−2^	2.40 × 10^−5^	5.25 × 10^−4^	0.06
Sf_inh_/[kg day mg^−1^]	6.3	12	–	–	–	–

## Results and discussion

### Geochemical and mineralogical properties

The massive sulfide ores in this region (inclusive of skarn, carbonate replacement, and vein-breccia ore types) are hosted on the early carbonate sequences and they are associated with Mesoproterozoic magmatic-hydrothermal activities (1.45~1.5 Ga) (Shen et al. 2018). The main characteristics of ore-hosting rocks in this area are consistent with those of hydrothermal sedimentary rocks proposed by Bostrom et al. (i.e., Fe/Ti ≥ 20, (Fe + Mn)/Ti ≥ 20, and Al/(Al + Fe + Mn) ≤ 0.35) (Boström 1983). The Pearson correlation matrix was used to analyze the correlation between trace metal elements in studied area (Table 4). Results show that there is a strong correlation among Cd (0.92), Zn (0.98), and Pb (0.91), which means they may have a common origin (Table 1). The correlation coefficients of Cu and Cd, Zn, and Pb are 0.63, 0.65, and 0.63, respectively (*P* ≤ 0.01), indicating that Cu has a certain correlation with the operation of the mining. This can be explained by the fact that the massive sulfide ore deposits in the studied area are characterized by sphalerite (ZnS), galena (PbS), pyrite (FeS_2_), and minor chalcopyrite (CuS), whereas the gangue minerals are dolomite (CaMg(CO_3_)_2_), calcite (CaCO_3_), quartz (SiO_2_), and slightly barite (BaSO_4_), rutile (TiO_2_), clay mineral, and so on (Dai et al. 2008). Geological analysis indicates that there are a large number of microdigitate stromatolites (MDS) in the sulfide deposit, which is mainly derived from the bio-precipitation of carbonate (Tang et al. 2013). Generally, MDS are developed in the sulfide deposit in Neoarchean-Paleoproterozoic successions but declined and gradually disappeared in Meso and Neoproterozoic carbonates. During this period, a frequent interaction occurred between microorganism and ions (Na^+^, K^+^, Cd^2+^, Zn^2+^, Pb ^2+^, Cl^−^, HS^2−^, and S^2−^) in seawater (Guo et al. 2013; VanLoon et al. 2013). In this way, the qualities of sulfide ores were improved through repetitive accretion of microbial mats, sediments, and/or entrapped inorganic materials.

**Table 4:** Pearson’s correlation matrix of trace metal elements in the farmland soils in the sphalerite mining area

	Cd	Cr	Cu	Hg	Pb	Zn	Mn	Fe
Cd	1							
Cr	− 0.38	1						
Cu	0.63**	0.13	1					
Hg	− 0.01	0.16	− 0.08	1				
Pb	0.92**	− 0.19	0.63**	− 0.13	1			
Zn	0.98**	− 0.35	0.65**	0.08	0.91**	1		
Mn	0.49	− 0.02	0.49	0.62*	0.36	0.56*	1	
Fe	0.40	0.11	0.43	0.76**	0.26	0.49	0.96**	1

*p < 0.05

**p < 0.01

**Fig. 2:**
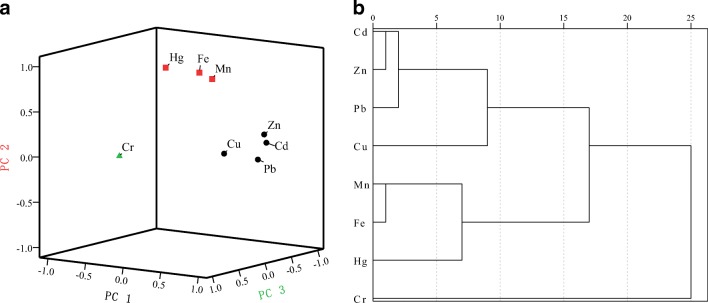
Multivariate statistical analyses of trace metals in the farmland soils from the three villages in the sphalerite mining area: **a** 3D plot of PCA loading for trace metal elements and **b** dendrogram of the trace metal elements obtained by hierarchical cluster analysis (single-linkage method)

The normalized rotated PCA and ANOVA were used to analyze the sources of trace metal elements in farmland soil. Table 5 indicates the rotational composition matrix of trace metal elements of the soil in the three villages around the mining area (PCA factor > 0.42 was set to italics). In the PCA analysis, the eight trace metal elements can be classified into three principal components (PCS) with respective eigenvalues of 3.60, 2.58, and 1.22 and a cumulative contribution of 92.35%. Figure 2a and b show a load curve graph of the PCS and the dendrogram of the trace metal elements obtained by hierarchical cluster analysis. On the basis of the above results, three groups of metal sources were identified by chemical and mineralogical patterns. In detail,

1. Group I represents obvious human contributions to trace metal elements correlated with mining operations. The first principal component (PC1) explains 44.94% of the variable information, in which Zn (0.91), Cd (0.92), Cu (0.83), Pb (0.94), and Mn (0.42) account for a large part of the factors. The differences of factors are related with the sedimentary environment and chemical components. Zn, Cd, Cu, and Pb are related to the mineralization. Specifically, Pb is related to galena, Zn and Cd are present in sphalerite, and Cu is mainly derived from the lesser proportion of chalcopyrite (CuS) in slag/ore. Furthermore, the presence of MDS indicates that there is an organic matter existing in the rocks, which is mainly derived from the interaction between ancient bacteria and cyanobacteria. The organic matter shows a certain correlation with the quality of the ore and the content of pyrite (FeS_2_). However, we did not pay much attention to this issue considering the complex process of biological evolution and geological movement.
2. Group II represents a mixing source (mining activities, rock, and soil elements). The loading rate of the second principal component (PC2) is 32.20%, and Mn (0.86), Fe (0.93), and Hg (0.92) are the dominant elements. The possible diagenetic elements Fe/Mn and the presence of Fe-Mn oxides in samples basically come from three major sources. Source one represents natural sources like Fe and Mn nodules in parent material of soils. However, Fe and Mn contents in the soil are not completely derived from parent materials. This is because the concentrations of Fe and Mn in the soil samples decrease as the distance between the sampling sites and the mine increases, which means that a fraction of Fe and Mn in the farmland comes from the mining activities. Therefore, sources two and three are essentially associated with the oxidation of pyrite (FeS_2_) and the weathering of nearby ore-hosting rock particles such as dolomite (CaMn(CO_3_)_2_) and calcite (CaFe(CO_3_)_2_) and shale. The geological analysis in this area supports this view (Solomatova and Asimow 2018), considering that the surrounding rocks of the massive sulfide ores are mainly the Manganese dolomite, iron-bearing dolomite, and black shale in the Great Wall (Li et al. 2003).
3. Group III represents natural sources (i.e., rock and soil elements). The third principal component (PC3) is lower, with Cr (0.95) representing 15.21%. Herein, the Cr values are slightly varied in the collected samples. Therefore, Cr is slightly influenced by mining operation and mainly derived from natural resources (i.e., clay minerals of soils).

**Table 5:** Rotated component for trace metal elements in the farmland soil around the contaminated plume surrounding the mining district (PCA factor loading> 0.42 is shown in italics)

Metals	PC1	PC2	PC3
Cd	*0.92*	0.16	− 0.29
Cr	− 0.16	0.08	*0.95*
Cu	*0.83*	0.13	0.37
Hg	− 0.20	*0.92*	0.01
Pb	*0.94*	0.01	− 0.11
Zn	*0.91*	0.26	− 0.27
Mn	*0.42*	*0.86*	0.02
Fe	0.31	*0.93*	0.11

### Trace metal pollution in soil and water resource

Table 6 shows the average concentrations of trace metals and pH values in mining tailings, soils adjacent to the tailings, topsoil of adjacent farmland, samples of mining drainage, and water samples collected in the spring and along the streams. The mineralization of this area is mainly composed of sphalerite (ZnS), pyrite (FeS_2_), and galena (PbS), which are the main sources of the high trace metal concentrations in the tailings and in the surrounding mining sites (Liu et al. 2013). The soil is mainly composed of sandy loam, with a pH ranging from 6.5 to 7.5.

**Table 6:** Concentrations of trace metals (means± standard deviation) and the pH ranges of the mine tailings, surface soils from the farmlands, and samples of mine drainage and stream water collected in the survey area

Sample type and location	pH	Cd	Cr	Cu	Hg	Pb	Zn	Mn	Fe
Tailings (mg kg^−1^ (*n* = 10))	–	31.1 ± 3.14	77.1 ± 7.23	302 ± 26.4	0.519 ± 0.046	2.86 × 10^3^ ± 174	7.65 × 10^3^ ± 940	4.46 × 10^3^ ± 252	4.77 × 10^4^ ± 1.67 × 10^3^
Soils adjacent to the tailings (mg kg^−1^ (*n* = 6))	–	20.6 ± 5.27	73.5 ± 8.42	278 ± 35.1	0.485 ± 0.062	1.70 × 10^3^ ± 257	5.04 × 10^3^ ± 807	3.98 × 10^3^ ± 304	4.11 × 10^4^ ± 1.25 × 10^3^
Mine drainage (mg L^−1^ (*n* = 3))	6.1–6.3	0.028 ± 0.011	0.061 ± 7.63 × 10^−4^	0.047 ± 2.74 × 10^−3^	0.118 ± 0.036	0.120 ± 9.62 × 10^−3^	12.0 ± 5.11	9.05 ± 5.18	–
Stream source water (mg L^−1^ (*n* = 2))	6.9–7.2	0.011 ± 2.70 × 10^−4^	0.053 ± 4.35 × 10^−4^	0.043 ± 2.37 × 10^−3^	0.110 ± 2.14 × 10^−3^	0.093 ± 2.03 × 10^−3^	0.273 ± 0.014	0.237 ± 0.013	–
Soils from village A (mg kg^−1^ (*n* = 20))	–	6.87 ± 0.22	69.5 ± 4.54	257 ± 32.5	0.406 ± 0.028	1.16 × 10^3^ ± 109	1.67 × 10^3^ ± 77.2	3.25 × 10^3^ ± 144	3.29 × 10^4^ ± 1.41 × 10^3^
Soils from village B (mg kg^−1^ (*n* = 20))	–	2.20 ± 0.16	81.3 ± 5.11	233 ± 15.4	0.446 ± 0.029	203 ± 20.6	485 ± 64.7	2.80 × 10^3^ ± 105	2.98 × 10^4^ ± 880
Soils from village C (mg kg^−1^ (*n* = 20))	–	1.59 ± 0.07	74.5 ± 5.32	216 ± 8.06	0.406 ± 0.031	194 ± 23.4	396 ± 41.8	2.44 × 10^3^ ± 98.8	3.11 × 10^4^ ± 760
Stream water (mg L^−1^ (*n* = 22))	6.3–7.0	0.015 ± 6.20 × 10^−4^	0.056 ± 4.01 × 10^−3^	0.041 ± 2.75 × 10^−3^	0.089 ± 0.012	0.099 ± 4.42 × 10^−3^	4.74 ± 0.804	3.55 ± 0.896	–
Ri^a^	–	1.40 ± 0.10	34.7 ± 5.79	35.3 ± 6.24	0.088 ± 0.009	43.6 ± 6.01	245 ± 25.9	630 ± 94.3	1.68 × 10^4^ ± 1.12 × 10^3^
Bi^b^	–	0.094	68.3	21.8	0.036	21.5	78.4	608	–
Si^c^	6.5–7.5	0.3	200	100	0.5	300	250	–	–
Wi^d^	6.0–9.0	0.01	0.1	1.0	0.001	0.1	2.0	–	–

^a^Regional background values

^b^Background concentrations of heavy metals in natural soils of Hebei province (SEPA 1990)

^c^Standards for heavy metal contents in the Soil Environmental Quality of China (SEPA 1995) grade II

^d^Standards for heavy metal contents in the Surface Water Environmental Quality of China (SEPA 2002) grade

The average content of Cd, Cr, Cu, Fe, Hg, Mn, Pb, and Zn in tailings of mining area is 31.1, 77.1, 302, 4.46 × 10^3^, 0.519, 4.77 × 10^4^, 2.86 × 10^3^, and 7.65 × 10^3^ mg kg^−1^. The concentrations of Cd, Cr, Cu, Fe, Hg, Mn, Pb, and Zn in soils adjacent to the tailing is 20.6, 73.5, 278, 3.98 × 10^3^, 0.485, 4.11 × 10^4^, 1.70 × 10^3^, and 5.04 × 10^3^, respectively (Table 6). Similar results have also been detected in other mining area. For example, Shu et al. (2018) found the averaged concentrations of trace metal elements in sulfide tailings of the Dabaoshan Mine are 2.54 × 10^3^ (Cu), 1.56 × 10^3^ (Zn), and 1.35 × 10^3^ (Pb) mg kg^−1^, respectively. Ordóñez et al. (2011) investigated three mercury mines in India. They found the averaged concentrations of Cd, Zn, Pb, and Cu are 0.43, 112, 43.9, and 60.8 mg kg^−1^, respectively.

The samples of the mine drainage show weak acidity (pH 6.1–6.3) and a high level of trace metal element pollution, with average concentrations for Cd, Cr, Cu, Hg, Pb, and Zn as high as 0.028, 0.061, 0.047, 0.118, 0.120, and 12.0 mg L^−1^, respectively (Table 4). The streams nearby the mining area also show high concentrations of trace metal elements and weak acidity (pH 6.2–6.5) (Table 6). However, their pH rapidly recovers to the neutral level (pH 6.8–7.0) and the trace metal element content also decreases significantly as the distance from the pollution source increases. The pH values of AMD in this area are similar with those obtained from Bhuiyan et al. (2010). Besides, results also show that a large amount of minerals carried in the stream migrates to the downstream farmland due to the slope of the mountains (Fig. 1). Nevertheless, the concentration of Cd in the streams is 1.5 times as high as the national standard of class V surface water, which means the surface water cannot meet the requirements for agriculture irrigation (SEPA 2002). The measured concentrations of Zn and Pb in water samples are much higher than those found at the Zeïda district (Morocco), where the metal concentrations were below the drinking water standards (El Azhari et al. 2017). Similar results were found in studies of other mining areas in Egypt (Redwan and Rammlmair 2016), India (Mohanty et al. 2018), Spain (Cánovas et al. 2016), and China (Wang et al. 2018).

The average concentrations of Cd, Cr, Cu, Hg, Pb, and Zn in the topsoil (sandy loam type) of the farmlands in the three villages were 3.55, 75.1, 235.33, 0.419, 850.33, and 57.9 mg kg^−1^, respectively. Thus, although the content of trace metal elements in farmland is much lower than that of tailings, it is still higher than the local background values (Zhang and Liu 2018). In detail, the average content of Pb and Zn in the two mining areas is as high as 240.01 and 612.04 mg kg^−1^, which is much higher than the local farmland soil (194.02 and 396.14 mg kg^−1^). The highest concentration of trace metal elements is found in the tailings of the middle of the hillsides, where the average content of Pb and Zn in the topsoil is as high as 732.5 and 3.023 × 10^3^ mg kg^−1^. This is because the terrain here is relatively flat, not promoting erosion severe processes. After decades, a large number of ores deposit in the surface soil with the help of rainfalls, with a maximum thickness of 3.2 cm. The concentrations of Zn and Pb in soil samples are much more variable among the sampling sites, with similar to the results of the Osor mine district (Bori et al. 2017), and 100 times higher than those found at the Zeïda district (Morocco) (El Azhari et al. 2017). Compared with the areas mentioned above, the trace metal pollution in soils and water caused by small mines in the Yanshan Mountains really deserves certain attention and vigilance.

The concentrations of Fe and Mn in the soil of the three villages are greater than that in the soil adjacent to the tailings. The maximum concentration of Mn and Fe is detected in village A, with the values of 3.25 × 10^3^ and 3.29 × 10^4^ mg kg^−1^, respectively. These results indicated that there exists natural accumulation of trace metal elements in the farmland adjacent to the mining areas (values are higher than geochemical background). Generally, the mining operations can account for the increasing of the concentrations of trace metal elements such as Cu, Zn, Hg, Cd, and Pb. Although the trace metal element concentrations in the farmland herein are not as high as that in the farmland surrounding several large-scale mines in the Hebei province, the soil pollution arising from small mining operation causes a profound impact on the ecological environment and is still worthy of attention.

**Fig. 3:**
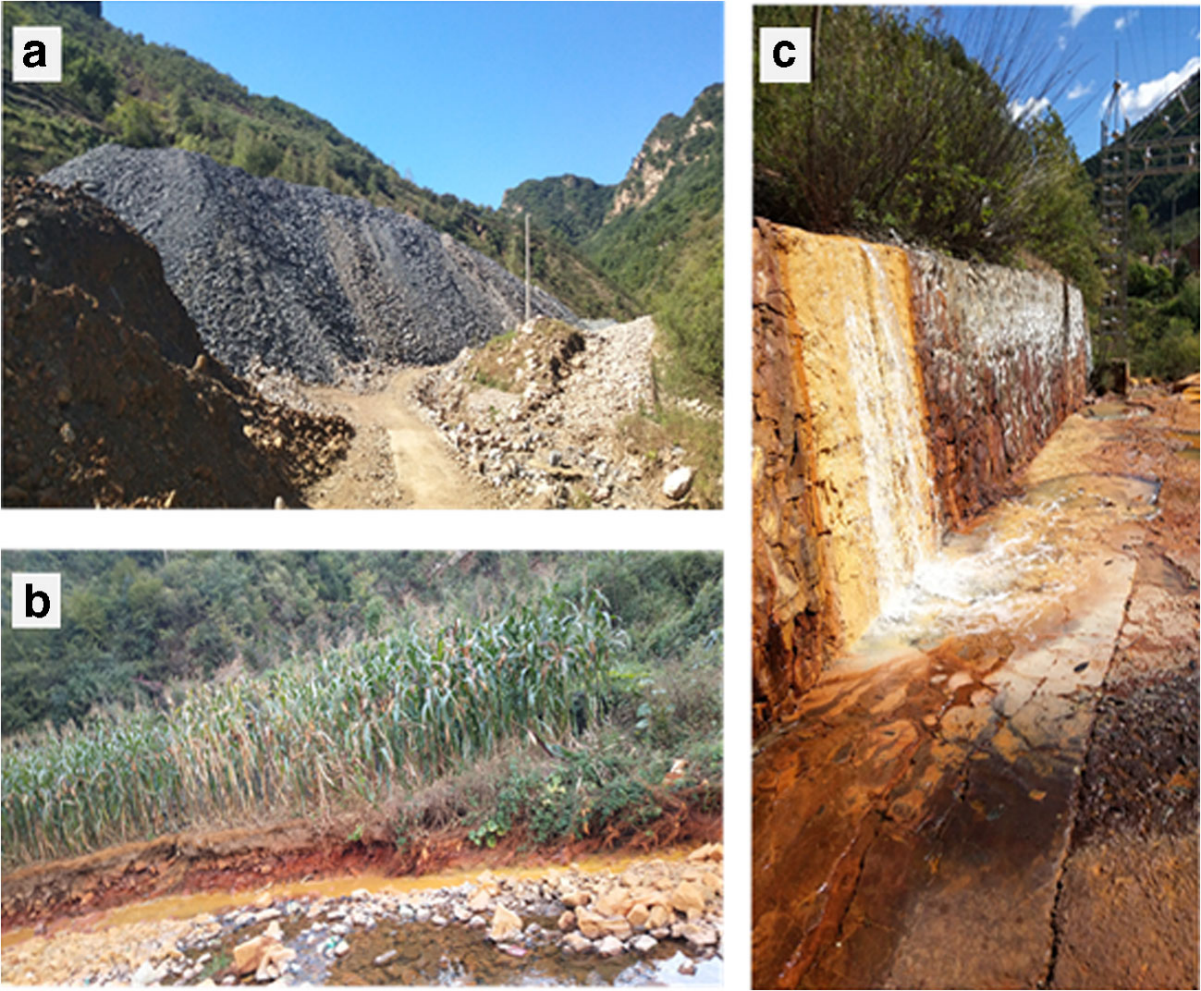
Photos taken at the mining district of the surveyed mine during August 2018. **a** Tails and bared soil. **b** Streams. **c** Mine drainage

**Fig. 4:**
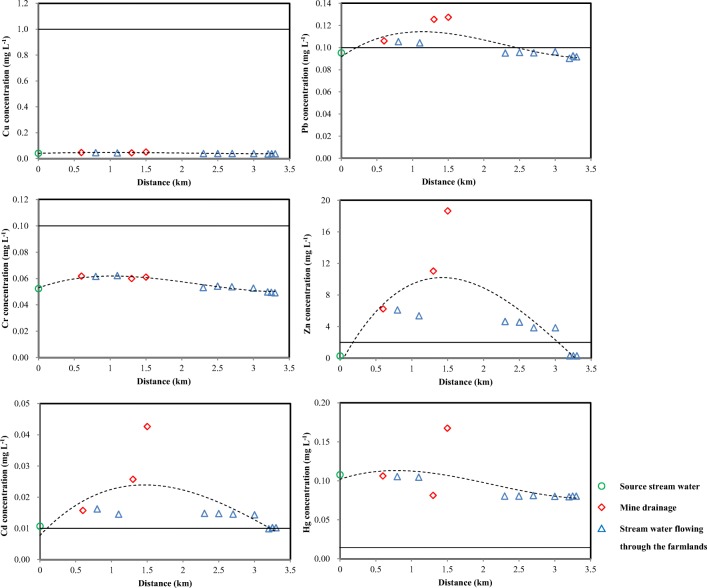
Changes in trace metal concentrations of the stream water as it flows away from the source in the sphalerite area. The straight lines represent the national standards for the trace metals in surface water of class V, which is applicable to the water bodies for agricultural use and landscape requirement (SEPA 2002)

Aspects of contamination dispersion from the mines are shown in Fig. 3, whereas in Fig. 4, it is indicated the common pollutant concentrations of Cu, Cr, Cd, Pb, Zn, and Hg in the streams nearby the mines, which gradually declined with the increase of the distance from the mining area (Fe and Mn are not included). Among others, usually mining activities lead to the accumulation and contamination of Cd, Cu, Pb, and Zn in the streams (Ali et al. 2016). These results show that the drainage of the abandoned mine still contains a high level of trace metal elements. The small particles of tailings delivered by the running streams will accumulate in the farmland far away from the mining area (Figs. 3 and 4). The migration of particles created from abandoned mining areas with convenient transportation is mainly correlated with natural factors (including wind, precipitation, surface runoff.) (Pourret et al. 2016; Rodríguez et al. 2009). Since the streams flow into the surface runoff through the tailings and then flow into the soil at the foot of the mountain (Nieva et al. 2018), interactions of the drainage coming from the mine can further contribute to the expansion of the flooded area, while rainfall events in summer may cause the release of a large quantity of trace metal elements transported from the mining source to adjacent farmland (Fig. 3). However, the concentrations of Cr and Hg in the sampling area are lower and less correlated with the distance, which can be explained by their geogenic source rather than the main mineralization.

**Fig. 5:**
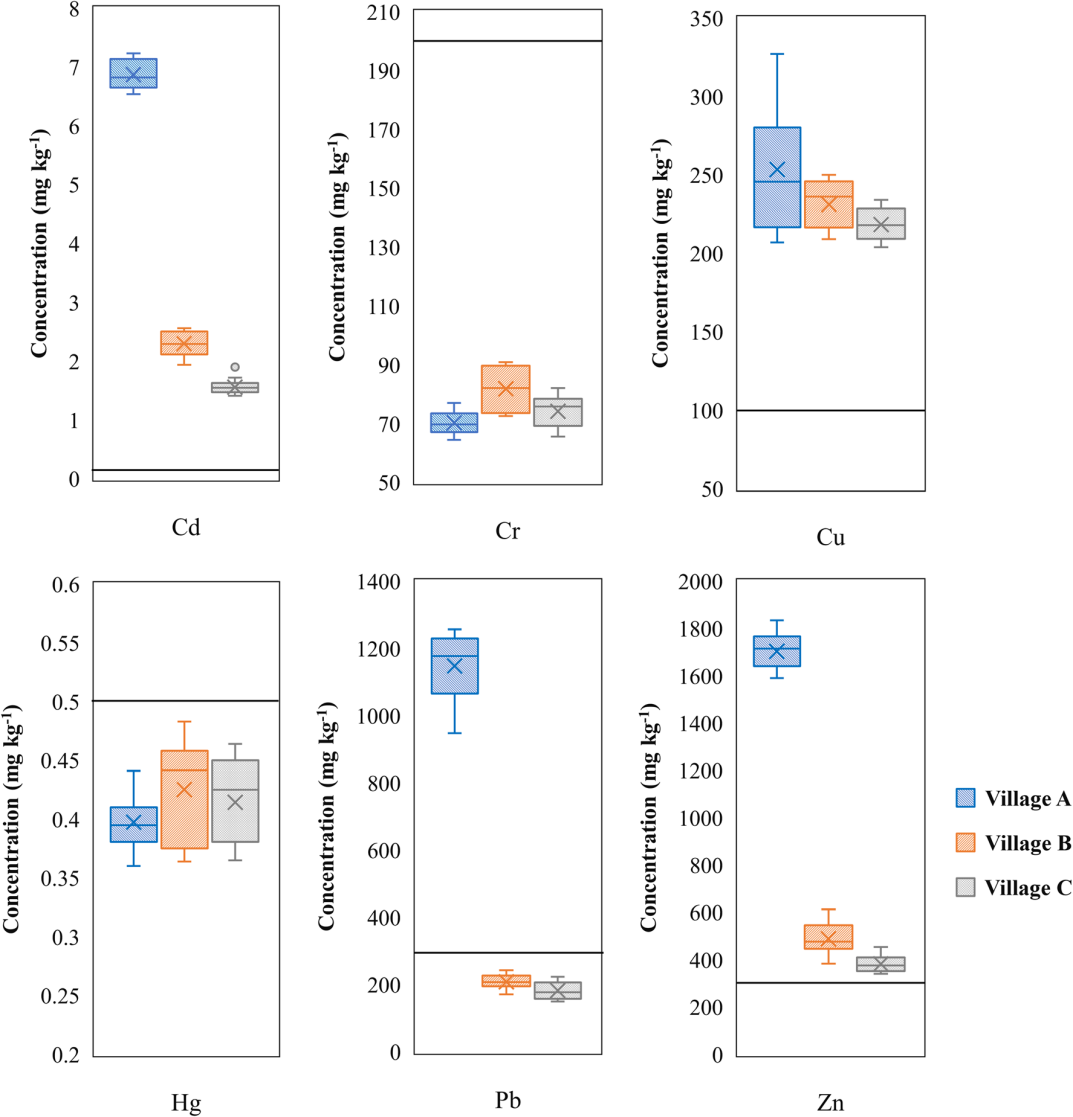
Box plots of Cu, Pb, Cr, Zn, Cd, and Hg concentrations in the surface soils from farmlands of the three villages. The straight lines indicate the grade II criteria (agricultural soil, pH > 6.5) of trace metals specified in the National Environmental Quality Standards for Soils in China (SEPA 1995)

Figure 5 compares the concentrations of common pollutants in the farmland of the three villages affected by mining operations (Fe and Mn are not included). The average concentrations of Cd, Cr, Cu, Hg, Pb, and Zn ranged as A > B > C. The reason for that can be inferred by the proximity of the village to the mine, while villages B and C are located relatively further. However, the concentrations of Cr and Hg in the surface layers of farmland in village A is lower than the standard of the local background values, despite the differences that were not significant. The trace metal elements in farmlands of villages B and C are directly affected by the drainage of the mine and related streams in the surrounding. Village B lies at the bottom of the basin area at the foot of the mountain, where the surface runoff can accumulate more trace metal elements, leading to higher content of those in the farmland. Although the farmland of village C is relatively further from the mining area, and the concentrations of Zn, Cd, and Pb is lower than that of villages A and B, the average concentrations of Cu, Zn, and Cd is higher than the national secondary standard (SEPA 1995). In addition, the small particles carried by the streams are easily deposited in the flat downstream basin due to rainfall (especially heavy rain). Consequently, the concentrations of trace metal elements in the surface layers of farmland (0–10 cm) in villages B and C increases.

### Pollution index and enrichment factors

Table 7 demonstrates the average single pollution index (Pi) and the corresponding Nemerow pollution index (PI_N_) for Cd, Cr, Cu, Hg, Pb, and Zn in the farmland’s topsoil of the three villages in the surveyed areas. On the basis of the Pi, the pollution caused by Cu, Zn, Pb, and Hg is moderately or slightly in the surface soil, while Cd reaches a serious extent. Figure 4 indicates that the content of Cu, Zn, and Cd, in approximately 80% of the soil samples, exceeds the class II standard (SEPA 1995). Meanwhile, the concentrations of Pb is also above that standard. According to the Pi (Table 7), the pollution degree of trace metal elements in farmland decreases as follows: Cd > Zn > Cu > Pb > Hg~Cr. The soil pollution of farmland in village C is the most serious, while the concentrations of Cr and Hg in the farmland are less affected by mining operations. Table S4 shows the EF values of the topsoil of the three villages. Among others, Cd is a typically enriched trace element in topsoil of the three villages (Table S4). Meanwhile, the average EF values of Zn, Pb, and Cu are significantly higher, revealing that they were highly accumulated in this area (Zhang and Liu 2018; Zhuang and Gao 2014).

**Table 7:** Mean pollution indices of Zn, Cd, Pb, and Cu in the surface soils of farmlands in the three villages of the mining area and the corresponding Nemerow pollution indices

Location	Single pollution index (Pi)						Nemerow pollution index (PI_N_)		
	Cd	Cr	Cu	Hg	Pb	Zn	Min	Mean	Max
A (*n* = 20)	23.53	0.37	2.86	0.93	4.13	7.02	0.32	6.47	7.00
B (*n* = 20)	7.85	0.43	2.47	0.93	0.74	2.21	0.45	2.44	8.27
C (*n* = 20)	5.50	0.40	2.23	0.86	0.70	1.72	0.37	1.90	9.07

### Potential ecological risks of trace metal elements

Table 8 demonstrates the individual risk index and the potential ecological risk index, *E*_*i*_ of the individual trace metal element, and the ecological risks associated with multi-trace metal elements (RI) in the topsoil of three villages in the mining area. Potential ecological risk caused by Cd, with a value over 500, is the greatest of all villages, followed by Hg (*E*_*i*_ > 400) (Table S2). Meanwhile, the same indices for Pb and Zn in village A remain at a high level, and those of Pb, Zn, and Cu in villages B and C normally stay at a moderate level. Furthermore, the *E*_*i*_ of Cr for all the villages is always below 2.5, which means a relatively low level (Table S2). The RI values indicate that the trace metal elements in the topsoil of the three villages may result in considerable potential ecological risks (He et al. 2018; Ren et al. 2016). On the other hand, the potential risks of the topsoil in the three villages have proven to decline in this sequence: A > B > C. It means that the more severe the farmland is polluted by trace metal elements, the greater the potential of ecological risk is. Nevertheless, Cd mainly from mining areas also poses potential ecological risks to certain species (Lu et al. 2018; Yu et al. 2016). The results show that the authorities not only need to enhance the regulation of mines in operation, but also need to properly handle abandoned mines and tailings, aiming at preventing the spread of trace metal elements to the environment.

**Table 8:** Potential ecological risk posed by individual trace metal element (Ei) and multiple trace metal elements (RI) in the farmland soils of the three villages surrounding the mining district based on the mean contents of trace metal elements

Location	Ei						RI
	Cd	Cr	Cu	Hg	Pb	Zn	
Village A (*n* = 20)	2192.01	2.01	58.86	451.23	269.50	213.73	3187.35
Village B (*n* = 20)	702.92	2.36	53.32	494.95	47.43	62.07	1363.04
Village C (*n* = 20)	506.41	2.16	49.53	451.23	45.21	50.68	1105.23

### Health risk assessment of surface trace metal elements

Table 9 is a comprehensive assessment of the non-carcinogenic health risks of local residents connected with trace metal element pollution caused by mining. The HI values showed that the non-carcinogenic health risks for children in the topsoil of the three villages were respectively 4.57 (A), 1.19 (B), and 1.11 (C), and for adults, the values were respectively 0.642 (A), 0.168 (B), and 0.157 (C). This revealed that there is no obvious non-carcinogenic health risk for adults in the area, but the non-carcinogenic health risk index for children is greater than the safety threshold. Similar with other studies, the impact of trace metal elements on local children’s health should not be ignored (Shen et al. 2016; Wei et al. 2015).

**Table 9:** Non-carcinogenic health risks in the farmland soils of the three villages surrounding the mining district based on the mean concentrations of trace metals

People	Heavy metals	HQ		
		Village A	Village B	Village C
Child	Cd	9.24 × 10^−2^	2.96 × 10^−2^	2.14 × 10^−2^
	Cr	2.96 × 10^−1^	3.47 × 10^−1^	3.18 × 10^−1^
	Cu	7.78 × 10^−2^	7.05 × 10^−2^	6.55 × 10^−2^
	Hg	1.66 × 10^−2^	1.82 × 10^−2^	1.66 × 10^−2^
	Pb	4.02	7.07 × 10^−1^	6.74 × 10^−1^
	Zn	6.75 × 10^−2^	1.96 × 10^−2^	1.60 × 10^−2^
	HI	4.57	1.19	1.11
Adults	Cd	1.33 × 10^−2^	4.28 × 10^−3^	3.08 × 10^−3^
	Cr	4.24 × 10^−2^	4.97 × 10^−2^	4.55 × 10^−2^
	Cu	1.09 × 10^−2^	9.88 × 10^−3^	9.18 × 10^−3^
	Hg	2.33 × 10^−3^	2.56 × 10^−3^	2.33 × 10^−3^
	Pb	5.64 × 10^−1^	9.92 × 10^−2^	9.45 × 10^−2^
	Zn	9.47 × 10^−3^	2.75 × 10^−3^	2.25 × 10^−3^
	HI	0.642	0.168	0.157

Table 10 shows the carcinogenic health risk index of trace metal elements Cd and Cr in the three villages. Both the single trace metal element carcinogenic risk index and the total cancer risk index are below the acceptable risk threshold recommended by US EPA, 10^−6^–10^−4^, demonstrating that the carcinogenic risk of trace metal elements in the areas is generally low and varies within the safe range, which will not cause carcinogenic risks in the population (Wang et al. 2016; Xu et al. 2016).

**Table 10:** Carcinogenic health risks in the farmland soils of the three villages surrounding the mining district based on the mean concentrations of trace metals

	Village A	Village B	Village C
*R*_*1*_(Cd)	3.91 × 10^−9^	1.25 × 10^−9^	9.02 × 10^−10^
*R*_*2*_(Cr)	7.51 × 10^−8^	8.80 × 10^−8^	8.07 × 10^−8^
*R*_total_	7.90 × 10^−8^	8.92 × 10^−8^	8.16 × 10^−8^

## Conclusions

This paper analyzed the pollution situation of small-scale sphalerite mines and evaluated health risk index caused by trace metal elements of the three villages in the Yanshan Mountains. The results show that (1) Pearson correlation matrix and principal component analysis (PCA) demonstrate that Zn, Cu, Cd, and Pb in soil are mainly derived from mining activities (including tailing dumping), and Cr is mainly contributed by natural sources. The content of trace metal elements in soil and water bodies decreases with the increase of the distance away from the mining areas, and the contamination caused by Zn, Cd, and Pb in the villages adjacent to the tailings is comparatively severer. (2) The pollution index and enrichment factor denote that the contamination of trace metal elements in farmland soil decreases in the order of Cd > Zn > Cu > Pb > Hg~Cr. The potential ecological risks of farmland in the three villages adjacent to the mines follow the order of A > B > C. In general, Cd-induced ecological risk is the severest. (3) The results of human health risk assessment indicate that the carcinogenic risks of trace metal elements in the three villages are 7.90 × 10^−8^ (A), 8.92 × 10^−8^ (B), 8.16 × 10^−8^ (C), respectively, which actually reflects no cancer risks. However, various pathways of exposure of trace metal elements in the mining areas pose obvious non-carcinogenic risks to local children, most significantly influenced by Pb.

Compared to the existing study efforts, this paper focused on the potential abilities of the abandoned small-scale mines to cause serious trace metal element pollution to adjacent areas. Especially, small particles from tailings and the leachate from tailings are likely to pollute nearby soil and surface water for several years. Nevertheless, there are still some drawbacks in this paper that need to be improved in the future researches. Firstly, the variations of trace metal element distribution in soils and water should be predicted with the help of geospatial technologies (i.e., GIS, Surfer, Mike SHE). Secondly, the study gives priority to address the ecological risks of trace metals caused by the mining activities, and the transport pathways of soil microorganisms, plants, and animals have not been thoroughly discussed. Thirdly, more remediation strategies, such as development of tailing ponds and use of geomembrane and phytoremediation measures, should be conducted to improve the ecological environment. Furthermore, the movement of trace metal elements from abandoned mining tailings should be effectively controlled and the risks to children should be cautiously reduced.

## Electronic supplementary material

ESM 1 (DOC 64 kb)
