# Supplementary material for: Trace metal element pollution of soil and water resources caused by small-scale metallic ore mining activities: a case study from a sphalerite mine in North China
Source: Environ Sci Pollut Res Int. 2019 Jun 25;26(24):24630–44. doi: 10.1007/s11356-019-05703-z (PMC6689288; doi:10.1007/s11356-019-05703-z)
Supplement: Supplementary file 1 — (DOC 64 kb) [file 11356_2019_5703_MOESM1_ESM.doc]

**Supplementary data** for

**Trace Metal Elements Pollution in Soil and Water Resources Caused by Small-scale Metallic Ore Mining Activities: A Case Study from a Sphalerite Mine in North China**

**Table S1**. Classification of soil pollution by trace metal elements based on the Nemerow pollution index (Nemerow 1991)

| Nemerow pollution index | Pollution level |
| --- | --- |
| <0.7 | Clean |
| 0.7-1.0 | Warning limit |
| 1.0-2.0 | Slightly polluted |
| 2.0-3.0 | Moderately polluted |
| >3.0 | Seriously polluted |

**Table S2**. Classification of potential ecological risk (Li et al. 2015) of soil trace metal elements pollution based on the risk factor and risk index (Hakanson 1980)

| Risk factor for single metal | Risk index for multiple metals | Ecological risk level |
| --- | --- | --- |
| <40 | 150 | Low |
| 40-80 | 150-300 | Moderate |
| 80-160 | 300-600 | Considerable |
| 160-320 | 600-1200 | High |
| >320 | >1200 | Very high |

**Table S3**. Classification of trace metal element enrichment in soils based on the enrichment factor (Sutherland 2000)

| Enrichment factor | Enrichment level |
| --- | --- |
| <2 | Deficiency to minimal |
| 2-5 | Moderate |
| 5-20 | Significant |
| 20-40 | Very high |
| > 40 | Extremely high |

**Table S4**. Values of enrichment factor for trace metal elements in the farmland soils of the three villages in the Sphalerite mining area

| Location | Trace metal element | Enrichment factor | | | |
| --- | --- | --- | --- | --- | --- |
|  |  | Mean | Max | Min | SD |
| Village A (*n*=20) | Cd | 89.18 | 93.97 | 84.88 | 2.90 |
|  | Cr | 28.60 | 32.45 | 24.01 | 2.09 |
|  | Cu | 20.60 | 22.19 | 19.08 | 0.91 |
|  | Hg | 1.24 | 1.41 | 1.13 | 0.08 |
|  | Pb | 1.45 | 1.61 | 1.30 | 0.09 |
|  | Zn | 1.33 | 1.50 | 1.18 | 0.09 |
| Village B (*n*=20) | Cd | 14.39 | 17.52 | 11.58 | 1.82 |
|  | Cr | 13.03 | 14.61 | 11.70 | 0.86 |
|  | Cu | 12.10 | 12.82 | 11.42 | 0.45 |
|  | Hg | 13.76 | 15.11 | 12.13 | 0.94 |
|  | Pb | 15.10 | 16.40 | 12.51 | 1.00 |
|  | Zn | 13.76 | 15.39 | 12.40 | 1.03 |
| Village C (*n*=20) | Cd | 65.63 | 74.73 | 52.43 | 6.21 |
|  | Cr | 11.55 | 13.62 | 9.82 | 1.17 |
|  | Cu | 11.01 | 12.77 | 8.57 | 1.33 |
|  | Hg | 25.98 | 28.59 | 24.29 | 1.20 |
|  | Pb | 7.55 | 9.52 | 6.02 | 1.01 |
|  | Zn | 6.16 | 7.20 | 5.15 | 0.65 |

**References**

Nemerow, N. L. C. (1974). Scientific stream pollution analysis. 210-231

Hakanson L (1980): An ecological risk index for aquatic pollution control.a sedimentological approach. Water Research 14, 975-1001

Li P, Lin C, Cheng H, Duan X, Lei K (2015): Contamination and health risks of soil heavy metals around a lead/zinc smelter in southwestern China. Ecotoxicol Environ Saf 113, 391-399

Sutherland RA (2000): Bed sediment-associated trace metals in an urban stream, Oahu, Hawaii. Environmental Geology 39, 611-627
